# Cervical range of motion and patient-reported outcomes in patients undergoing surgical treatment for degenerative pathology of the subaxial cervical spine (C3–C7)

**DOI:** 10.1007/s10072-026-09346-0

**Published:** 2026-08-26

**Authors:** Claudio Cornali, Claudia Riva, Francesco Belotti, Emma Sala, Marco Mazzali, Nicola Pagani, Simona Serioli, Riccardo Bergomi, Marco Maria Fontanella, Luca Zanin

**Affiliations:** 1 Department of Medical and Surgical Specialties, Radiological Sciences and Public Health, University of Brescia, Brescia, Italy; 2 Unit of Neurosurgery, ASST Spedali Civili di Brescia, Brescia, Italy; 3 SC Medicina del Lavoro, Igiene, Tossicologia e Prevenzione Occupazionale, ASST Spedali Civili di Brescia, Brescia, Italy; 4 Department of Medical and Surgical Specialties, Radiological Sciences and Public Health, Unit of Occupational Health and Industrial Hygiene, University of Brescia, Brescia, Italy; 5 Department of Neurosurgery, Papa Giovanni XXIII Hospital, Bergamo, Italy; 6 Technology for Health PhD Program, University of Brescia, Brescia, 25124 Italy; 7 Department of Medical and Surgical Specialties, Unit of Neurosurgery, Radiological Sciences and Public Health, University of Brescia, Largo Spedali Civili 1, 25123 Brescia, Italy

**Keywords:** Subaxial cervical spine, Range of motion, Degenerative cervical disease, Neck disability index, Patient-reported outcomes, Surgical treatment

## Abstract

**Background:**

Degenerative subaxial cervical (C3–C7) disease often requires surgery when conservative care fails, yet the relationship between post-operative cervical range of motion (ROM) and patient-reported outcomes is poorly defined. We evaluated post-operative ROM and its cross-sectional association with clinical outcomes.

**Methods:**

In a retrospective cohort with cross-sectional outcome assessment, 100 patients operated between 2015 and 2021 (anterior or posterior approach, with or without instrumentation) were assessed at a single follow-up visit. Neck-specific disability (Neck Disability Index, NDI), health-related quality of life (Short Form-12, SF-12) and cervical ROM (CROM device) were measured; pre-operative ROM was unavailable. Spearman and partial Spearman correlations (adjusted for age and comorbidity) and Mann–Whitney U comparisons were used.

**Results:**

Median NDI was 16% (IQR 8–35), in the minimal-disability band; the SF-12 physical component (PCS-12, 40.2 ± 10.0) was below and the mental component (MCS-12, 47.6 ± 11.1) near the population norm of 50. NDI correlated negatively with ROM in all planes and with total ROM (ρ = −0.46, *p* < 0.001), and PCS-12 positively (axial rotation ρ = +0.52, *p* < 0.001); MCS-12 showed no significant association. Associations persisted after adjustment and in a Pearson sensitivity analysis. Exploratory comparisons, confounded by case-mix, showed greater axial rotation after the anterior approach.

**Conclusions:**

Greater post-operative ROM is associated with lower neck-specific disability and better physical quality of life, independent of age and comorbidity, but not with mental quality of life. The cross-sectional design precludes inference about the direction of these associations. Prospective studies with pre-operative ROM baselines are needed.

## Introduction

Degenerative pathology of the cervical spine encompasses a spectrum of age-related conditions that narrow the cervical canal and compress the spinal cord and nerve roots [1, 2]. The underlying lesions are heterogeneous. They include congenital canal stenosis, intervertebral disc degeneration with osteophyte formation (“hard disc”) or disc protrusion (“soft disc”), and hypertrophy of the laminae, facet joints or ligaments. They also include subluxation from disc and facet degeneration, altered mobility associated with severe spondylosis, and deformities such as loss of the physiological cervical lordosis [3].

The global prevalence of cervical spine degeneration is substantial, affecting up to 90% of individuals over 65 years of age [3]. Cervical degeneration is a primary cause of neck pain, with symptoms becoming increasingly common between 40 and 60 years of age; although radiological signs are present in most individuals over 50 years old, only a minority manifest clinical symptoms [4, 5].

The pathophysiology is multifactorial, involving mechanical, systemic, environmental and genetic influences [6]. Non-modifiable risk factors include age, sex, family history, genetic factors, general health status, coexisting low back pain and previous trauma; modifiable factors include sustained postural head flexion, psycho-social and behavioural factors, smoking and a sedentary lifestyle [7].

The subaxial cervical spine (C3–C7) is highly mobile relative to other spinal regions [8]. This mobility derives from the intervertebral discs together with the paired zygapophyseal (facet) and uncovertebral joints, and from the relatively horizontal orientation of the cervical facets, which permits a wide range of flexion, extension, lateral bending and axial rotation essential for head and neck function; the triangular vertebral foramen accommodates the spinal cord but does not itself generate motion [8]. It should be noted, however, that axial rotation is not distributed uniformly across the cervical spine: approximately half — and by some in-vivo estimates the majority — of total cervical axial rotation is generated at the atlanto-axial (C1–C2) segment, above the subaxial levels addressed in this cohort [9, 10].

Surgical treatment is indicated to prevent neurological deterioration [11, 12]. For cervical radiculopathy and axial pain, surgery is generally considered after conservative measures have failed — immobilisation with a soft collar, activity modification, non-steroidal anti-inflammatory drugs, corticosteroids, opioids and physiotherapy [3, 13]. For moderate-to-severe degenerative cervical myelopathy, by contrast, current guidelines recommend timely surgical decompression rather than a mandatory trial of conservative care, because delay risks irreversible deterioration [1, 11, 12].

Assessment of clinical outcomes after cervical spine surgery is essential for evaluating treatment efficacy and guiding decision-making. Validated tools include the Neck Disability Index (NDI), which measures neck-related disability [14], and the Short Form-12 (SF-12), which assesses health-related quality of life. Measurement of cervical ROM provides complementary information on the biomechanical function of the cervical spine after surgery [15].

Despite the importance of these measures, the relationship between cervical ROM and clinical outcomes after surgery for degenerative cervical pathology remains poorly characterised. Clarifying it could illuminate the biomechanical correlates of clinical outcome and potentially inform surgical decision-making.

The present study evaluated the cross-sectional relationship between post-operative cervical ROM and clinical outcomes, measured by the NDI and SF-12, in patients who underwent surgery for degenerative pathology of the subaxial cervical spine (C3–C7). We additionally compared outcomes between surgical approaches (anterior versus posterior) and by instrumentation status. We hypothesised that greater post-operative ROM would be associated with better clinical outcomes.

## Methods

### Study design and ethical approval

This retrospective cohort study with cross-sectional outcome assessment was conducted at a tertiary referral neurosurgical centre and is reported according to the Strengthening the Reporting of Observational studies in Epidemiology (STROBE) guidelines [16]. The study was approved by the local research ethics committee (protocol NP 1471, approved version of 19 April 2023); all patients provided informed consent for the research use of their clinical data. The study adhered to the Declaration of Helsinki.

### Patient selection

Patients who underwent surgical treatment for degenerative pathology of the subaxial cervical spine (C3–C7) between 2015 and 2021 were eligible. Inclusion criteria were: (1) age ≥ 18 years; (2) degenerative cervical pathology of the subaxial spine (cervical spondylosis, disc herniation, spinal stenosis or myelopathy); (3) surgery via an anterior or posterior approach; and (4) a minimum of 12 months from surgery to assessment. Exclusion criteria were previous cervical spine surgery, cervical trauma or fracture, cervical infection or tumour, inflammatory rheumatic disease of the cervical spine, congenital cervical abnormalities, a documented history of major psychiatric disorder (given its potential to influence self-reported outcomes, particularly the SF-12), and incomplete clinical data.

### Clinical assessment tools

Neck Disability Index (NDI). The NDI is a patient-reported measure of neck-related disability comprising 10 items each scored 0–5, expressed as a percentage, with 0–20% indicating minimal disability, 21–40% moderate, 41–60% severe, 61–80% crippling and 81–100% complete disability [14]. The validated Italian version was administered.

Short Form-12 (SF-12). The SF-12 yields a Physical Component Summary (PCS-12) and a Mental Component Summary (MCS-12), each normalised in the original US standardisation to a mean of 50 and standard deviation of 10, with higher scores indicating better health [17]. The validated Italian version was administered; scores were interpreted against the US-standardised norm, and benchmarking against Italian normative values is acknowledged as preferable in the Limitations.

Range of motion (ROM). Cervical ROM was measured with a cervical range of motion (CROM) device in three planes: sagittal (flexion and extension), coronal (right and left lateral bending) and axial (right and left rotation) [18]. The patient moved the head maximally without pain. ROM in each plane was defined as the sum of its two directional measurements and total ROM as the sum of all six directional measurements. Three trained examiners performed the CROM measurements. Each movement was recorded as the mean of three consecutive trials, and the examiners were blinded to the patients’ questionnaire scores. Pre-operative ROM was not available; post-operative values are reported descriptively and compared qualitatively with published CROM-derived healthy-adult norms [19]. For reference, healthy-adult CROM values reported by Youdas et al. [19] approximate 46° flexion, 67° extension, 62° axial rotation and 37° lateral bending to each side; all six motions decline significantly with age, so the age-matched comparator for the present cohort (mean age at follow-up 60.6 years) lies below these adult means.

Charlson Comorbidity Index (CCI). Comorbidity was quantified with the age-adjusted CCI [20, 21], which adds the weighted comorbidity score of Charlson et al. [20] to one point for each decade of age above 40 years, as proposed in the 1994 validation [21]. Because the raw (non-age-weighted) comorbidity score was not separately recorded, a de-aged comorbidity score was reconstructed by subtracting the age component (one point per decade above 40 years) from the age-adjusted index; age and this de-aged score were then entered as separate covariates in adjusted analyses to avoid collinearity. This reconstruction is acknowledged as non-standard.

### Surgical procedures

Patients underwent an anterior or posterior approach, with or without instrumentation, according to the pathology, the number of levels involved and the surgeon’s preference. Anterior procedures comprised anterior cervical discectomy and fusion (ACDF) with intervertebral cage or bone graft, and anterior cervical corpectomy and fusion (ACCF) with mesh cage or structural allograft, with plate-and-screw fixation where used. Posterior procedures comprised laminectomy, laminoplasty and foraminotomy, with lateral mass or pedicle screw fixation where used. In this cohort all 28 anterior or combined procedures and 61 of the posterior procedures involved instrumented fusion or fixation (89 patients, 89%), whereas the remaining 11 posterior procedures were non-instrumented decompressions (laminectomy or laminoplasty).

### Follow-up protocol

Patients were assessed at a single follow-up visit in 2022–2023 (mean time from surgery 55.8 ± 20.7 months; range 17–92). At this visit they completed the NDI and SF-12 and underwent CROM measurement. Radiographic evaluation comprised anteroposterior, lateral and flexion–extension views to assess stability and fusion status.

### Statistical analysis

Analyses were performed with SPSS 25.0 (IBM Corp., Armonk, NY, USA). Because the principal variables — the NDI in particular — were non-normally distributed and bounded, the primary analyses were non-parametric. Continuous variables are summarised as median (interquartile range, IQR) and, for comparison with prior literature, also as mean ± standard deviation; categorical variables as frequencies and percentages. Associations between ROM and clinical outcomes (NDI, PCS-12, MCS-12) were assessed with Spearman rank correlation, and partial Spearman correlations additionally adjusted for age and the de-aged comorbidity score; Pearson correlations were computed as a sensitivity analysis. Because some movements could not be recorded in every patient, correlations were computed on all available pairs and the sample size is reported for each coefficient. Between-group comparisons (anterior versus posterior; instrumented versus non-instrumented) used the Mann–Whitney U test, given non-normal distributions and markedly unequal group sizes. No formal correction for multiple comparisons was applied; correlation results are interpreted as associative and the subgroup comparisons as exploratory, with nominal significance thresholds (*p* < 0.05) read conservatively.

## Results

### Patient demographics and baseline characteristics

Of 187 patients operated for degenerative subaxial cervical (C3–C7) disease during the study period who met the eligibility criteria, 132 were invited to a dedicated research follow-up visit and 100 attended and were analysed (response rate 75.7%). The mean age at surgery was 56.0 ± 11.7 years and the mean age at follow-up 60.6 ± 12.0 years; 60 patients were men and 40 were women. A posterior approach was used in 72 patients (72%), an anterior approach in 25 (25%) and a combined approach in 3 (3%). Surgery involved a single level in 50%, two levels in 31%, three levels in 16% and more than three levels in 3%. Eighty-nine patients (89%) underwent instrumented fusion or fixation — all 28 anterior or combined procedures and 61 posterior procedures — while the remaining 11 underwent non-instrumented posterior decompression (laminectomy or laminoplasty). The mean age-adjusted CCI was 2.5 ± 1.7, indicating a relatively low comorbidity burden. Baseline characteristics are summarised in Table 1.

**Table 1:** Patient demographics and baseline characteristics

Characteristic	Value
Total patients, n	100
Age at surgery, years	56.0 ± 11.7
Age at follow-up, years	60.6 ± 12.0
Time from surgery, months	55.8 ± 20.7 (17–92)
Men / women, n	60 / 40
Age-adjusted Charlson Comorbidity Index	2.5 ± 1.7
Approach — posterior / anterior / combined, n	72 / 25 / 3
Levels — 1 / 2 / 3 / >3, %	50 / 31 / 16 / 3
Instrumented fusion or fixation, n (%)	89 (89.0)
– anterior or combined	28
– instrumented posterior	61
Non-instrumented posterior decompression, n (%)	11 (11.0)

Data are mean ± standard deviation (range) or number (percentage). The instrumentation variable distinguishes instrumented fusion/fixation from non-instrumented decompression; a separate arthrodesis-versus-motion-sparing (e.g. laminoplasty) classification was not coded. *CCI* Charlson Comorbidity Index

### Range of motion and clinical outcomes

Post-operative ROM could be recorded in 94–100 patients depending on the movement. Median total ROM was 197.0° (IQR 152.0–238.3; mean 199.9 ± 67.1), and median values by plane were 68.9° (flexion–extension), 87.3° (axial rotation) and 43.2° (lateral bending). These values are lower than published CROM-derived healthy-adult norms [19]; however, because no pre-operative ROM was available, the proportion of this reduction attributable to surgery rather than to pre-existing degeneration and age cannot be determined, and the comparison is descriptive only. Median NDI was 16% (IQR 8.0–34.5; mean 21.4 ± 17.9), within the minimal-disability band; the right-skewed distribution (mean exceeding median) reflects a subgroup with moderate or greater disability. The SF-12 physical component (PCS-12) was 40.2 ± 10.0 (median 41.5), below the US-standardised norm of 50, whereas the mental component (MCS-12) was 47.6 ± 11.1 (median 50.2), close to it. Directional and summary values are reported in Table 2.

**Table 2:** Post-operative cervical range of motion and clinical outcomes

Variable	Mean ± SD	Median (IQR)	*n*
*Range of motion (°)*			
Flexion	36.1 ± 20.3	36.8 (23.4–46.4)	98
Extension	33.8 ± 15.8	32.2 (22.0–42.9)	97
Right rotation	41.2 ± 18.7	43.0 (26.2–55.0)	95
Left rotation	43.5 ± 16.7	44.9 (30.4–54.9)	94
Right lateral bending	22.7 ± 13.2	20.8 (14.0–28.1)	100
Left lateral bending	22.9 ± 10.4	21.9 (15.3–29.6)	100
Flexion–extension (plane)	69.9 ± 31.4	68.9 (48.6–88.7)	96
Axial rotation (plane)	84.6 ± 29.4	87.3 (67.6–104.1)	94
Lateral bending (plane)	45.5 ± 19.2	43.2 (32.5–57.9)	100
Total ROM	199.9 ± 67.1	197.0 (152.0–238.3)	91
*Patient-reported outcomes*			
NDI (%)	21.4 ± 17.9	16.0 (8.0–34.5)	100
SF-12 PCS-12	40.2 ± 10.0	41.5 (31.7–48.3)	100
SF-12 MCS-12	47.6 ± 11.1	50.2 (39.0–56.7)	100

Each plane is the sum of its two directional measurements and total ROM the sum of all six; plane and total values are computed on patients with complete measurements for the constituent movements and may therefore differ marginally from the sum of the directional values. NDI 16% (median) lies within the minimal-disability band; PCS-12 is below and MCS-12 close to the US-standardised norm of 50. No pre-operative ROM baseline was available; values are descriptive. *IQR* interquartile range, *NDI* Neck Disability Index, *SF-12* Short Form-12, *PCS* Physical Component Summary, *MCS* Mental Component Summary, *ROM* range of motion

### Correlation between range of motion and clinical outcomes

**Fig. 1:**
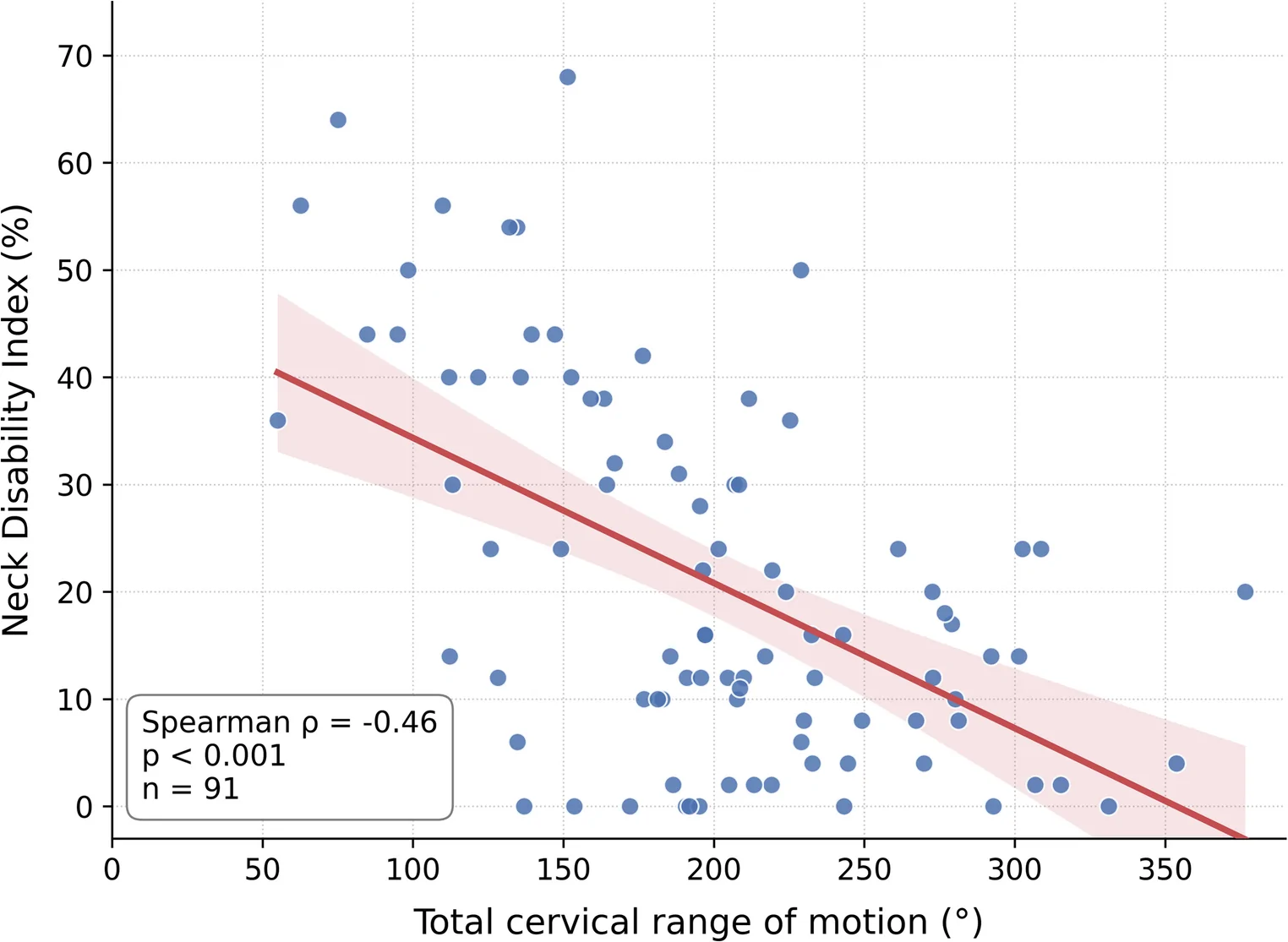
Scatter plot of total post-operative cervical range of motion (ROM, sum of the six directional measurements) against the Neck Disability Index (NDI) in 91 patients with complete data. Greater total ROM was associated with lower neck-specific disability (Spearman ρ = −0.46, *p* < 0.001). The solid line is the ordinary least-squares fit (Pearson *r* = − 0.52) and the shaded band its 95% confidence interval, shown as a visual guide. Because the assessment was cross-sectional, the direction of the association cannot be inferred

By Spearman rank correlation, the NDI correlated negatively with ROM in all planes — most strongly for axial rotation (ρ = −0.437, *p* < 0.001) and flexion–extension (ρ = −0.381, *p* < 0.001), and more weakly for lateral bending (ρ = −0.279, *p* = 0.005) — and with total ROM (ρ = −0.459, *p* < 0.001, *n* = 91), indicating that greater ROM was associated with lower disability (Table 3; Fig. 1). The PCS-12 correlated positively with ROM, most strongly for axial rotation (ρ = +0.521, *p* < 0.001) and flexion–extension (ρ = +0.370, *p* < 0.001). The MCS-12 showed no significant association with ROM in any plane. All correlations persisted after adjustment for age and the de-aged comorbidity score (Table 3) and were materially unchanged in a Pearson sensitivity analysis (e.g. total ROM versus NDI: Pearson *r* = − 0.52 versus Spearman ρ = −0.46), confirming robustness to the choice of correlation method.

**Table 3:** Spearman correlations between range of motion and clinical outcomes

Outcome	Model	Flexion–extension ρ (*p*)	Axial rotation ρ (*p*)	Lateral bending ρ (*p*)
NDI	Unadjusted	−0.381 (< 0.001)	−0.437 (< 0.001)	−0.279 (0.005)
NDI	Adjusted	−0.403 (< 0.001)	−0.460 (< 0.001)	−0.311 (0.002)
PCS-12	Unadjusted	+ 0.370 (< 0.001)	+ 0.521 (< 0.001)	+ 0.295 (0.003)
PCS-12	Adjusted	+ 0.356 (< 0.001)	+ 0.504 (< 0.001)	+ 0.265 (0.008)
MCS-12	Unadjusted	+ 0.193 (0.060)	+ 0.121 (0.245)	+ 0.125 (0.216)
MCS-12	Adjusted	+ 0.161 (0.121)	+ 0.021 (0.845)	+ 0.093 (0.364)

Values are Spearman rank correlation coefficients (*p*-value). Adjusted models are partial Spearman correlations controlling for age and the de-aged Charlson comorbidity score. For NDI, negative coefficients indicate that greater ROM is associated with lower disability; for SF-12, positive coefficients indicate that greater ROM is associated with higher (better) scores. n ranged from 94 to 100 across coefficients. A Pearson sensitivity analysis yielded materially identical conclusions. Correlation strength: weak (<0.30), moderate (0.30–0.70), strong (>0.70). *NDI* Neck Disability Index, *PCS* Physical Component Summary, *MCS* Mental Component Summary, *ROM* range of motion

### Comparison between surgical approaches and by instrumentation

Exploratory between-group comparisons are reported in Table 4. Anterior and posterior groups differed significantly only in axial rotation (98.1 ± 22.3° vs. 81.1 ± 30.0°, *p* = 0.009); the difference in PCS-12 favouring the anterior approach did not reach statistical significance (43.7 ± 9.8 vs. 39.2 ± 10.0, *p* = 0.052), and the remaining parameters did not differ. These comparisons are strongly confounded by case-mix: the anterior group was predominantly single-level (19/25, 76%), whereas the posterior group was predominantly multilevel (42/72 had two or more levels). Non-instrumented patients (*n* = 11), who were predominantly treated at three levels (6/11), had higher NDI (33.6 ± 22.1% vs. 19.8 ± 16.8%, *p* = 0.036) and lower ROM than instrumented patients; these differences likewise reflect the more complex pathology that led to a non-instrumented multilevel decompression rather than an effect of instrumentation itself. None of the nominally significant subgroup differences would survive correction for the number of comparisons performed and are reported as hypothesis-generating.

**Table 4:** Outcomes by surgical approach and by instrumentation status

Parameter	Posterior (*n* = 72)	Anterior (*n* = 25)	*p*	No instr. (*n* = 11)	Instr. (*n* = 89)	*p*
SF-12 PCS-12	39.2 ± 10.0	43.7 ± 9.8	0.052	36.5 ± 11.8	40.6 ± 9.8	0.190
SF-12 MCS-12	47.6 ± 10.9	48.6 ± 11.8	0.575	44.8 ± 11.3	47.9 ± 11.1	0.421
NDI score (%)	22.6 ± 18.5	16.4 ± 15.2	0.157	33.6 ± 22.1	19.8 ± 16.8	0.036*
Flexion–extension (°)	67.8 ± 27.4	80.4 ± 37.3	0.130	53.3 ± 23.5	72.1 ± 31.7	0.044*
Axial rotation (°)	81.1 ± 30.0	98.1 ± 22.3	0.009*	65.0 ± 26.3	87.2 ± 28.9	0.017*
Lateral bending (°)	46.1 ± 19.6	45.1 ± 18.2	0.977	37.0 ± 19.6	46.6 ± 19.0	0.134

Data are mean ± standard deviation; *p*-values are from the Mann–Whitney U test. *Nominal *p* < 0.05, none of which survives correction for multiplicity. The three combined-approach patients are excluded from the approach comparison. ROM denominators vary slightly (n = 94–100 overall) because some movements could not be recorded in every patient. Comparisons are unadjusted and confounded by the number of levels treated (see text). *NDI* Neck Disability Index, *PCS* Physical Component Summary, *MCS* Mental Component Summary, *ROM* range of motion

## Discussion

This study evaluated the cross-sectional relationship between post-operative cervical range of motion and clinical outcomes after surgery for degenerative pathology of the subaxial cervical spine. The principal finding is that greater post-operative ROM was associated with lower neck-specific disability and better physical health-related quality of life, independent of age and comorbidity, whereas mental quality of life was not meaningfully related to ROM.

### Range of motion after surgery

Post-operative cervical ROM in this cohort was lower than CROM-derived values reported in healthy adults across all planes [19]. This is expected, since both the underlying degenerative disease and the surgical treatment reduce mobility: the majority of patients (89%) underwent instrumented fusion or fixation, which limits segmental motion, whereas only the 11 non-instrumented patients had motion-preserving or purely decompressive posterior procedures. In the absence of a pre-operative baseline, the present data cannot apportion the observed reduction between pre-existing degeneration, ageing and the operation, and the normative comparison is descriptive only.

### Range of motion and clinical outcomes

The moderate negative correlation between ROM and the NDI, and the positive correlation between ROM and the PCS-12, indicate that patients who retained greater cervical mobility tended to report less neck-related disability and better physical functioning. The strongest associations were with axial rotation, a movement central to everyday activities such as driving and therefore particularly salient to patient-perceived function. This finding must be interpreted in light of cervical rotational biomechanics. Because the CROM device records global head-on-trunk motion, the measured axial rotation reflects the summed contribution of all cervical segments, and in the normal spine a large proportion of rotation is generated at the atlanto-axial (C1–C2) joint rather than at the operated subaxial levels [8–10]. The association between global axial rotation and outcome should therefore be read as a relationship between overall neck function — which remains functionally relevant to activities such as driving irrespective of its segmental origin — and self-reported status, not as evidence of restored motion at the operated segments. Any difference in measured axial rotation between subgroups nonetheless localises to the subaxial spine, since the atlanto-axial joint is unaffected by subaxial surgery; but because segmental contributions were not quantified (e.g. by dynamic CT), a mechanistic interpretation of the rotation-specific findings is not warranted. The absence of an association with the MCS-12 indicates that cervical mobility relates to the physical rather than the psychological dimension of quality of life, consistent with the constructs of the NDI and the SF-12 physical component.

### Directionality and the cross-sectional design

A central interpretive caveat is that outcomes and ROM were assessed at a single time point, so the direction of the observed associations cannot be established. Active ROM measured with the CROM device is effort- and pain-dependent: a patient with greater pain or disability may guard movement and so produce a lower recorded ROM, meaning that higher disability could be a cause rather than a consequence of reduced motion. The reverse pathway — preserved mobility contributing to better function — is equally compatible with these data. This ambiguity would not be resolved by adding a pre-operative ROM measurement, because the correlation of interest is between post-operative ROM and concurrent outcome; only a longitudinal design with repeated measures could disentangle the temporal relationship. The present results therefore identify ROM as a functional correlate of outcome, not as evidence that surgically preserving motion improves outcome.

### Surgical approach and instrumentation

The exploratory subgroup analyses must be interpreted with particular caution. The greater axial rotation and the non-significant difference in physical quality of life favouring the anterior group, and the worse disability and ROM in the small non-instrumented group, are confounded by the number of levels treated and by the indication for surgery: anterior procedures were performed predominantly for single-level focal disease, whereas posterior and non-instrumented procedures were performed predominantly for multilevel stenotic myelopathy. Case-mix, rather than the approach or instrumentation per se, is the most plausible explanation, and this interpretation is consistent with pooled individual-patient-data analyses showing that outcome differences between anterior and posterior surgery for degenerative cervical myelopathy are largely attributable to baseline and disease characteristics rather than to the approach itself [22]. No causal inference regarding the superiority of any approach or of instrumentation can be drawn, and the small non-instrumented subgroup (*n* = 11) further limits any comparison.

### Clinical implications

The association between preserved mobility and better physical outcomes is hypothesis-generating only. Whether it has any bearing on the selection of motion-preserving strategies in appropriately chosen patients with focal pathology cannot be determined from a cross-sectional study that included no such procedures; the choice of operation must continue to be dictated by the pathology, the need for decompression and the requirement for stability.

### Limitations

Several limitations should be acknowledged. First, the retrospective, single-centre design and the absence of a pre-operative ROM baseline preclude any quantification of surgically induced change and, as discussed, any inference about the direction of the ROM–outcome relationship. Second, outcomes were assessed only in patients who attended a voluntary research visit a median of several years after surgery; such attenders are a self-selected subset, and selection bias could distort both the distributions and the observed correlations if, for example, more symptomatic or conversely more mobile patients preferentially returned. A comparison of attenders with non-attenders, which the available data did not permit, would be required to quantify this risk. Third, ROM and the patient-reported outcomes were assessed at the same follow-up visit; although the examiners performing the CROM measurements were blinded to the questionnaire scores and each movement was recorded as the mean of three consecutive trials — mitigating observer and single-measurement bias — concurrent assessment still precludes inference about the direction of the association, as discussed above. Fourth, the dataset distinguished instrumented fusion/fixation from non-instrumented decompression but did not separately code arthrodesis versus motion-sparing procedures such as laminoplasty, so instrumentation is only an approximate proxy for fusion. Fifth, the specific technique, the operated levels and the surgeon were not modelled, and the subgroup comparisons are consequently confounded by indication. Sixth, multiple correlations and comparisons were performed without formal correction, so nominally significant findings should be interpreted conservatively, and the small non-instrumented subgroup yields low power. Seventh, the comorbidity adjustment relied on an age-adjusted index that was de-aged by subtraction; residual confounding cannot be excluded. Finally, the SF-12 was interpreted against the US standardisation rather than Italian normative values, which may shift the absolute interpretation of the component scores. Reassuringly, the primary Spearman analyses and the Pearson sensitivity analysis were concordant, indicating that the central associations are robust to the choice of statistical method.

### Future directions

Prospective studies incorporating pre-operative and serial post-operative ROM measurements, together with longitudinal patient-reported outcomes, are needed to clarify the temporal relationship between cervical mobility and clinical outcome. Such studies should report complete participant flow, adjust for the number of levels treated, the specific pathology and the surgical technique, and could directly compare motion-preserving and fusion-based strategies in matched populations.

## Conclusion

In this cohort of patients treated surgically for degenerative pathology of the subaxial cervical spine, greater post-operative cervical range of motion was associated with lower neck-specific disability and better physical quality of life, independent of age and comorbidity, whereas mental quality of life was unrelated to mobility. Because outcomes were assessed cross-sectionally, the direction of these associations cannot be established. Post-operative ROM may nonetheless serve as a useful functional correlate of clinical outcome, and prospective studies with pre-operative baselines and longitudinal follow-up are required to determine whether preserving cervical mobility translates into measurable clinical benefit.

## Data Availability

The datasets generated and/or analysed during the current study are not publicly available due to institutional data-protection requirements but are available from the corresponding author on reasonable request.
